# Correction: Enantioselective synthesis of isochromans and tetrahydroisoquinolines by C–H insertion of donor/donor carbenes

**DOI:** 10.1039/d0sc90081h

**Published:** 2020-05-06

**Authors:** Leslie A. Nickerson, Benjamin D. Bergstrom, Mingchun Gao, Yuan-Shin Shiue, Croix J. Laconsay, Matthew R. Culberson, Walker A. Knauss, James C. Fettinger, Dean J. Tantillo, Jared T. Shaw

**Affiliations:** Chemistry Department, University of California, Davis One Shields Ave Davis CA 95616 USA jtshaw@ucdavis.edu

## Abstract

Correction for ‘Enantioselective synthesis of isochromans and tetrahydroisoquinolines by C–H insertion of donor/donor carbenes’ by Leslie A. Nickerson *et al.*, *Chem. Sci.*, 2020, **11**, 494–498, DOI: 10.1039/C9SC05111B.

The electron pushing arrows in Fig. 6B were, unbeknownst to the authors, converted by ChemDraw (version 18.0) into double-headed resonance arrows during the final stages of galley proof review and went unnoticed until the article appeared in print. This event was traced to a version-specific bug in the software that has been resolved in a subsequent update. A corrected figure is provided here.

**Fig. 1:**
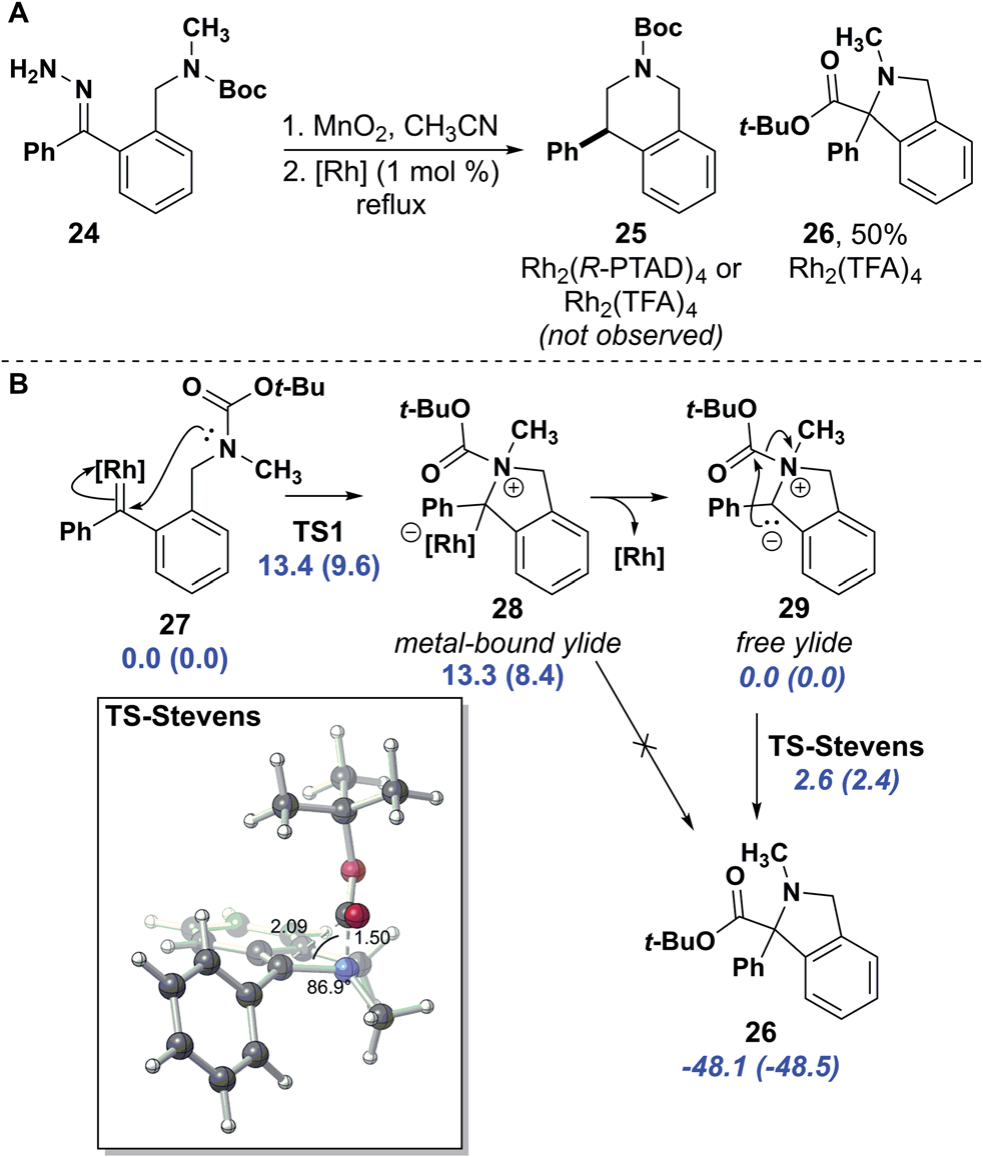
(A) Stevens rearrangement product synthesis. (B) The DFT (uB3LYP/LANL2DZ[6-31G(d)]) computed mechanism suggests that *N*-attack to the rhodium carbene and the subsequent Stevens rearrangement is energetically feasible at experimental conditions; relative free energies (electronic energies in parentheses) for metal-bound (normal text) and ylide (*italics*) reactions are reported in kcal mol^−1^.

The Royal Society of Chemistry apologises for these errors and any consequent inconvenience to authors and readers.

